# Core self-evaluations, psychological capital, social comparison, and socioeconomic status: an analysis of Generation Z’s satisfaction in the post-pandemic era

**DOI:** 10.3389/fpsyg.2026.1665300

**Published:** 2026-07-22

**Authors:** Xin Yan, Yu-Shan Chen, Yu-Ning Lin

**Affiliations:** 1 School of Finance and Accounting, Fuzhou University of International Studies and Trade, Fuzhou, Fujian, China; 2 Department of Business Administration, National Taipei University, Taipei, Taiwan; 3 School of Environment, Tsinghua University, Beijing, China

**Keywords:** academic satisfaction, core self-evaluations, Generation Z, life satisfaction, psychological capital, social comparison, socioeconomic status

## Abstract

**Introduction:**

In the post-pandemic era, the world has witnessed profound transformations with economic, social, and lifestyle changes. In such a special period, Generation Z is also undergoing unique trials. While confronting multifaceted and complex challenges, they also have positive adaptations, including improved psychological capital (PSY) and core self-evaluations (CSE). As the life and academic satisfaction for Generation Z have become an increasingly critical issue for higher education, we wanted to seek new insight into the reasons which influence the life and academic satisfaction for Generation Z. Previous studies have suggested that both CSE and PSY were separately positive predictors of young people’s sense of happiness. However, the indirect relationship between CSE and Generation Z’s satisfaction via PSY has rarely been researched.

**Methods:**

Based on positive psychology, social cognition theory, and social comparison theory, we develop a double moderated-mediation model and empirically tested our hypotheses using questionnaires from 753 Chinese college students, representing the Chinese youth. We further investigated PSY as a mediator and social comparison as well as socioeconomic status as double moderators in the relationships between CSE and Generation Z’s satisfaction.

**Results:**

Our study indicated that low social comparison and high socioeconomic status could strengthen the positive effect of CSE on Generation Z’s satisfaction in daily life. Especially, CSE and PSY play essential roles in students’ mental state.

**Discussion:**

The in-depth exploration of the complex relationships among these factors will help schools and families develop more effective strategies to improve the satisfaction and mental health of Generation Z in the long run.

## 1 Introduction

Generation Z, who were the children of Generation X or millennials, includes people born from the mid-to-late 1990s to the early 2000s. Being known as “digital natives,” members of Generation Z are digitally literate, well-schooled, self-restraint, and responsive, and the life and academic satisfaction for Generation Z have become increasingly critical issues for higher education. Previous research has proved that life satisfaction is an important resource which college students could rely on and plan for their future lives (Abdullah et al., 2022). Variations of satisfaction models have been tested for university students in China (Gu and Lu, 2023), as well as in other developing countries. Since higher education is extremely important in traditional Chinese culture, and academic achievement count for a great deal in Generation Z’s college life (Guo et al., 2023), life satisfaction was found to be linked with academic participation especially in recent studies (Antaramian, 2017; Stavrulaki et al., 2020).

In the post-epidemic era, Generation Z in China still encounter many difficulties: first, the economic downturn has led to fierce competition in the job market, and the phenomenon of “lying flat” in Generation Z has increased; second, the popularity of individualized entertainment such as live broadcasts and short videos has gradually replaced traditional campus culture, which increases the loneliness of college students; third, due to hardware and network conditions, students in poor areas have poor online learning effects, exacerbating educational inequality. All of these have affected the life satisfaction and academic satisfaction of Chinese college students of Generation Z.

In support of Positive Psychology, our findings emphasize the critical role of psychological capital (PSY), which could make Generation Z more confident, or braver when they face difficulties in post-pandemic era. Psychological capital refers to a positive and proactive mental state typified by efficacy, hope, optimism, and resilience (Luthans et al., 2007). At the same time, psychological quality and emotions are also very important to young people’s learning. While PSY was proved to be related to college students’ well-being, little attention was paid to understanding how core self-evaluations (CSE) and PSY interact and affect college students’ satisfaction, Harris et al. (2023) has tested the interrelationship between self-esteem and optimism, which were part of both CSE and PSY, respectively. However, the relationship between the three (satisfaction, CSE, and PSY) was still unclear and previous findings were more speculation-based than empirical demonstrated.

Specifically, based on social comparison theory (Festinger, 1954), our second aim is to exam the moderating role of social comparison. Skovgaard-Smith et al. (2020) suggested that many people always exclude those who are inferior to themselves in order to improve their self-identity. Most people would unconsciously compare themselves with those around them, and with the advancement of the Internet and the popularization of social software such as Instagram and WeChat, this comparison has become ubiquitous among adults or even adolescents. While in the Chinese society, greater academic achievement is believed to be associated with a brighter future, the ranking and comparison of academic scores could also affect Generation Z’s life and academic satisfaction. Researches about social comparison have emerged during recent years, however, little has combined social comparison with PSY, CSE, life satisfaction, and academic satisfaction. Consequently, we propose that social comparison a great influence on people’s daily life, it is also important in understanding how CSE via PSY influences students’ life and academic satisfaction. Our study calls attention to the role of social comparison, which is a fundamental part of being human.

In addition to the above-mentioned aims, this study investigates socioeconomic status as a moderator for the relationship between CSE and PSY. Existing theories and researches indicated that socioeconomic status has more or less affected people’s happiness in life, but scholars have rarely considered the moderating role of socioeconomic status between Generation Z’s CSE and PSY, which is a substantial gap in the previous literature. Therefore, we propose that socioeconomic status also plays a critical role in understanding how CSE via PSY impacts Generation Z’s life and academic satisfaction.

Additionally, our model is important to consider from the perspective of Generation Z, which are the representatives of Chinese youth. It is of vital importance to explore the factors which affected their life and academic satisfaction in post-pandemic era and strengthening the mental health of the younger generation may prove to be pivotal in a country.

## 2 Theoretical foundation and research hypotheses

### 2.1 Theoretical foundation

This research is based on diverse theoretical perspectives to explain how CSE contribute to better life and academic satisfaction via PSY. More specifically, Positive psychology stands as the main theoretical foundation, it provides the basic logic for understanding how CSE and PSY translate into human well-being. Based on social comparison theory and social cognitive theory, we also put forward that social comparison and socioeconomic status could influence the indirect relationship between CSE and Generation Z’s satisfaction via PSY. In summary, this study uses positive psychology as its core theoretical foundation, integrating social comparison theory and social cognitive theory to jointly construct and explain the research framework, and systematically explore the influence mechanisms among these variables.

#### 2.1.1 Positive psychology

Developed by Seligman (1998), positive psychology teaches people to seek a better life, and explains people’s psychology and behavior of pursuing happiness with scientific methods. It encourages people to emphasize happiness and positivity so that the state of one’s mind might change from negative to positive. In support of Positive Psychology, this study contributed a new perspective for both CSE and PSY.

#### 2.1.2 Social cognitive theory

Social cognitive theory explores a dynamic interrelationship among environment, people, as well as their behaviors and it developed by Bandura (1986), self-efficacy is one’s confidence to meet the challenges and influence behavior through its effects on expected outcomes (Bandura et al., 1999). People with poor self-efficacy always have a suspicion that they cannot do anything well, and always flinch and escape when they encounter difficulties.

#### 2.1.3 Social comparison theory

Developed by Festinger (1954), social comparison theory indicates that individuals are inclined to weigh their own social worth based on whether they are superior to others. People cultivate their self-motivation, self-improvement, and a positive self-image by comparing themselves to others across domains such as wealth, intelligence, attractiveness, and success (Festinger, 1954). Social comparison is a popular psychological phenomenon, everyone wants to know his status, ability, and level unconsciously by comparing with others.

### 2.2 Core self-evaluations, life satisfaction, and academic satisfaction

Core self-evaluations (CSE) was first conceptualized by Judge (1997). It refers to an individual’s holistic and basic evaluation of their own abilities and value, with four dimensions including generalized self-efficacy, neuroticism, locus of control, and self-esteem. Judge et al. (2003) develop a classic measure, with a total of 12 items. CSE has been widely used since its introduction. Shen et al. (2021) suggested CSE was related to strategic decision-making and has great potential providing leverage effects to researchers on executive self-concept. CSE is closely linked with personal work success (Loignon and Scheaf, 2025), person-job fit (Zhang and Yan, 2024), work engagement (Ma et al., 2022), even job burnout (Peng et al., 2016) and academic burnout (Paloș, 2023).

The life satisfaction and academic satisfaction of Generation Z are both closely related to their mental well-being and long-term growth, and are of vital importance (Nguyen et al., 2025; Zhu et al., 2025). Life satisfaction refers to a comprehensive and cognitive evaluation of an individual’s life based on self-determined criteria (Diener et al., 1985). Academic satisfaction is considered as students’ general sentiment and evaluation of their educational experiences, scholastic environment, learning performance and academic goals (Lent et al., 2005).

### 2.3 Mediating effects of psychological capital

Psychological capital (PSY) refers to a positive psychological condition that an individual exhibit during their personal growth process, it includes four aspects: efficacy, hope, optimism, and resilience (Luthans et al., 2007). Recent studies acknowledged that both PSY and CSE were separately positive predictors of well-being (Saleem et al., 2022; Bertieaux et al., 2024). On the one hand, CSE has a positive effect on students’ life satisfaction (Broucek, 2005), on the other hand, previous research indicated that better PSY is bound up with lower burnout and higher career satisfaction of nurses (Ali and Ali, 2014). People with better PSY will be more enthusiastic about their work, leading to a higher sense of life happiness (Karatepe and Karadas, 2015). In addition, Larson and Luthans (2006) reveal that PSY can be used to interpret the ideal attitude of people. Students with better PSY might also feel more life-satisfied and academic-satisfied when facing academic challenges and difficulties. Drawing upon the core tenets of positive psychology, we propose that PSY serves as the critical psychological mechanism linking personality traits to outcomes.

Previous researches indicated that CSE and PSY were both important for life satisfaction. In the light of social cognitive theory, students with higher CSE are more inclined to perceive themselves as capable and agentic, which directly fosters the development of psychological capital. However, it is still unclear how CSE and PSY interact and affect Generation Z’s satisfaction. Some scholars found the existence interacts between hope and self-restraint, while other academics suggested the relationship between tenacity and self-efficacy in some similar studies, however, there is still a lack of systematic and complete analysis on the impact of CSE on PSY. As a solution, the focus of our research is the mediating role of PSY, through which we exploring a new way linking CSE to generation Z’s satisfaction. We used the samples from college students in mainland China, representing the generation Z of China.

Based on the abovementioned discussion, we made the following assumptions:

*Hypothesis 1*: The positive relationship between CSE and life satisfaction is mediated by PSY.

*Hypothesis 2*: The positive relationship between CSE and academic satisfaction is mediated by PSY.

### 2.4 The moderated mediation effects of social comparison

Social comparison (SC) is considered as the process by which individuals evaluate their own capabilities, perceptions, and properties by comparing themselves with others (Festinger, 1954). Generation Z is the generation of high-tech products and social networks, they have been greatly influenced by the Internet from childhood. The advent of the Internet has brought people many conveniences, however, more and more Generation Z perceive social networking apps such as Facebook and WeChat as sources of both attraction and depression. Social networking apps provide platforms for Lee (2022), which caused a lot of discontentment (Meier and Johnson, 2022). Not only adults, but also the children’s life satisfaction are affected due to social comparison (Ruble and Frey, 1991). Parents believe that education and examination are the most effective ways to achieve career success for their children in China, and it caused increased competition and social comparison among Chinese students. However, numerous studies have demonstrated that social comparison can elicit negative emotions, thereby affecting mental health and life satisfaction (McCarthy and Morina, 2020; Lee, 2022; Meier and Johnson, 2022).

However, few studies have combined social comparison with PSY, CSE, and generation Z’s satisfaction. Social comparison theory suggests that social comparison acts as an environmental stressor that interferes with the positive translation of personal traits (CSE) into psychological resources (PSY). Therefore, we assume that strong social comparison would reduce one’s self-efficacy and self-esteem, which are part of CSE, and thereby weakening the impact of CSE on PSY. Moreover, we assume that strong social comparison would affect Generation Z’s optimism and hope, which are part of PSY, and thereby weakening the indirect relationship between CSE and life satisfaction via PSY. That is to say that the indirect relationship from CSE via PSY to Generation Z’s life and academic satisfaction will be weaker when their social comparison is stronger. Our research aims to find out if social comparison moderates the proposed relationship between CSE and PSY, and whether social comparison affects the indirect relationship from CSE via PSY to Generation Z’s life and academic satisfaction. Therefore, we made the following assumptions:

*Hypothesis 3*: The relationship between CSE and PSY is negatively moderated by social comparison (3a). In addition, the indirect relationship between CSE and life satisfaction via PSY is negatively moderated by social comparison such that the indirect relationship becomes stronger as social comparison is weaker (3b), the indirect relationship between CSE and academic satisfaction via PSY is negatively moderated by social comparison such that the indirect relationship becomes stronger as social comparison is weaker (3c).

### 2.5 The moderated mediation effects of socioeconomic status

Socioeconomic status (SES) is the most important and frequently examined sociological and psychological variable. Socioeconomic status (SES) is conceptualized as an individual’s relative position in the socioeconomic hierarchy, it mainly measured by economic resources, educational level, and occupational position (Adler and Ostrove, 1999). It indicates one’s position in the social structure according to social and economic factors. Regarding the issue of socioeconomic status on people’s psychological state and well-being, three perspectives (positive, negative, and neutral) have been formed. Some scholars believed that children’s optimism is closely related to their parents’ education level (Hu et al., 2024) while others believed that people with lower socioeconomic status could have worse mental states (Kardash et al., 2023). Early studies such as Ulbrich et al. (1989) investigated the stress responses of black and white people with low socioeconomic status. They found that black people with low socioeconomic status tend to be affected by undesirable events, and less likely to be affected by economic crises than their white counterparts. However, Ge (2017) found that differences in socioeconomic status had no significant effect on children’s happiness. Some findings suggest the existence of moderating effects of socioeconomic status, but few literatures have directly explained the moderating role of socioeconomic status between CSE and PSY. Based on the abovementioned discussion, we assume that lower socioeconomic status will reduce Generation Z’s self-esteem and self-confidence, which is part of CSE, and thereby weakening the impact of CSE on PSY. We also assume that lower socioeconomic status will also cool Generation Z’s optimism and hope, which are part of PSY, thus weakening the indirect relationship between CSE and both life and academic satisfaction via PSY. Therefore, we make the following assumptions:

*Hypothesis 4*: The relationship between CSE and PSY is positively moderated by socioeconomic status (4a). In addition, the indirect relationship between CSE and life satisfaction via PSY is positively moderated by socioeconomic status such that the indirect relationship becomes stronger as socioeconomic status is higher (4b); the indirect relationship between CSE and academic satisfaction via PSY is positively moderated by socioeconomic status such that the indirect relationship becomes stronger as socioeconomic status is higher (4c).

**Figure 1:**
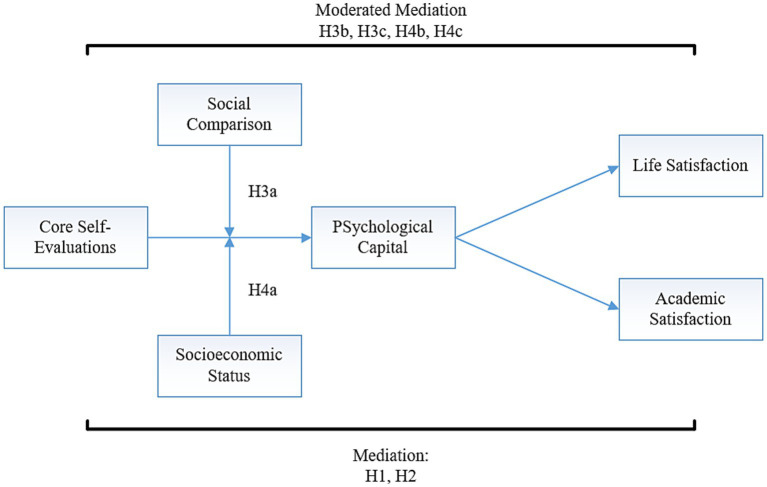
Research framework.

The researcher proposes the following framework as shown in Figure 1.

## 3 Methodology

### 3.1 Samples and procedures

This study focuses on universities located in the southeastern coastal region of China, including universities directly under the Ministry of Education, key and specialized provincial universities, and private universities. It covers multiple disciplines such as economics, education, literature, science, engineering, management, and arts. We solicited participants for this study by contacting the students’ unions. A total number of 3,512 questionnaires were distributed. Of these, college students from more than 80 classes participated in the survey, including those transferring from junior colleges to bachelor’s programs, undergraduates, master’s students, and doctoral students, representing different schools, grades, and majors. These students of different ages, as core participants in campus activities, have diverse and profound experiences with the main constructs of the questionnaire. Our questionnaire explains these abstract research concepts and translates them into their real feelings about daily campus life. The questionnaire items are mainly taken from mature scales published in authoritative journals and adapted to the characteristics of Chinese university students. The questionnaire underwent rigorous expert validity review, with university experts reviewing the content and wording of the questions. A pre-test was then conducted, and the question wording was optimized based on feedback from students in the pre-test, ensuring high reliability and validity.

To reduce common method bias, the questionnaire employed several procedural measures, including student anonymity and the use of a certain number of reversed items. The questionnaire used a mature scale that focused on describing students’ real satisfaction with their learning and life, rather than subjective and extreme views with personal biases. The researchers distributed the questionnaires personally in universities and explained the purpose of this survey to every student who completed the questionnaire and ensured the anonymity and confidentiality of the data. One thousand one hundred seven questionnaires were collected, representing a 31.5% response rate. The non-participation rate (68.5%) was primarily due to the principle of voluntary participation of the study or the heavy academic workload. A systematic screening procedure was applied to the collected responses. A total of 354 cases (32%) were excluded based on the following reasons: (1) Incomplete responses containing missing values; (2) Invariant response patterns, where the same scale option was selected throughout the answers. The final sample was 753.

### 3.2 Measures

#### 3.2.1 Core self-evaluations

Core self-evaluations (CSE) are an individual’s overall and fundamental assessment of their own abilities and value in various aspects (Judge, 1997). In this study, we adopted the classic measure Core Self-Evaluations Scale developed by Judge et al. (2003) to evaluate Generation Z’s CSE. It is a 5-point Likert scale used in a number of studies (Piccolo et al., 2005). The scale comprises 12 items spanning four dimensions, with sample items such as “I am confident I get the success I deserve in life.” In this study, the CR for the total scale was 0.927.

#### 3.2.2 Psychological capital

Psychological capital (PSY) refers to a positive and proactive mental state typified by efficacy, hope, optimism, and resilience (Luthans et al., 2007). This study employed the psychological capital questionnaire scale developed by Luthans et al. (2007). Sample items were: “I feel confident analyzing a long-term problem to find a solution.” Participants responded on a 5-point Likert scale (1 = “strongly disagree,” 5 = “strongly agree”). The CR for the total scale was 0.957.

#### 3.2.3 Social comparison

Social comparison (SC) is defined as an automated cognitive process by which individuals evaluate their own abilities, viewpoints, and traits by comparing themselves with others (Festinger, 1954). We utilized a scale developed by Gibbons and Buunk (1999) with 11 items, 5-point Likert scale to measure social comparison. Example item includes “I often compare how my loved ones are doing with how others are doing.” The CR of the scale in this study was 0.921.

#### 3.2.4 Life satisfaction

Life satisfaction (LS) refers to a holistic and cognitive judgment of an individual’s life based on self-defined criteria (Diener et al., 1985). It is a core concept for measuring an individual’s psychological well-being. We used SWLS questionnaire developed by Diener et al. (1985) to measure participants’ life satisfaction. The SWLS is a globally recognized measure for capturing the cognitive component of subjective well-being. This 5-point Likert scale includes 5 items. Sample items include “I am satisfied with my life.” The CR of this scale was 0.960.

#### 3.2.5 Academic satisfaction

Academic satisfaction (AS) is considered as students’ subjective evaluation and emotional acceptance of their educational experiences, campus environment, learning outcomes and interest experiences (Lent et al., 2005). Academic satisfaction was measured using a 5-point scale developed by Lent et al. (2005), including 7 items. Sample items include “I enjoy the level of intellectual stimulation in my courses.” The CR of this scale was 0.933.

#### 3.2.6 Socioeconomic status

Socioeconomic status (SES) refers to an individual’s relative position in the socioeconomic hierarchy; it was mainly determined by economic, educational, and occupational conditions (Adler and Ostrove, 1999). A 4-item scale of social status developed by Hollingshead (2021) was applied in this study to measure the socioeconomic status of the students’ families. Parental education and occupation were used in this study. Parental education was categorized into five levels, ranging from 1 (primary school or below) to 5 (graduate degree). Parental occupation was classified into five levels based on professional prestige and skill requirements, ranging from 1 (unskilled laborers) to 5 (professional administrators). Finally, the total scores of the father and mother are averaged to create a comprehensive socioeconomic status score for each Generation Z student.

## 4 Results

### 4.1 Descriptive statistics

Most participants who responded were aged between 16 and 25 (99.9%, n = 752). Male students accounted for 19%, and female students accounted for 81%. Among those students, 5.2, 27.1, and 27.2% of the respondents came from the first, second, and third-tier cities in China, respectively. 40.5% of the respondents came from fourth-tier cities or rural areas.

### 4.2 Correlation analysis

Descriptive statistics (gender and age) and correlation matrix are shown in Table 1, CSE was positively related to PSY as we expected (*r* = 0.61, *p* < 0.001), life satisfaction (*r* = 0.37, *p* < 0.001), and academic satisfaction (*r* = 0.37, *p* < 0.001). PSY correlated strongly with life satisfaction (*r* = 0.49, *p* < 0.001) and academic satisfaction (*r* = 0.54, *p* < 0.001). Life satisfaction and academic satisfaction were positively related to each other (*r* = 0.64, *p* < 0.001).

**Table 1:** Means, standard deviations, reliabilities, and correlations among study variables.

Variable	Mean	S. D.	1	2	3	4	5	6	7	8
1. Gender	1.81	0.39	–							
2. Age	2.50	0.50	−0.03	–						
3. CSE	3.27	0.52	−0.13***	−0.03	(0.93)					
4. PSY	3.53	0.46	−0.16***	0.00	0.61***	(0.96)				
5. SC	3.22	0.48	−0.12***	−0.05	−0.12***	0.17***	(0.92)			
6. SES	27.43	13.26	−0.17***	−0.11**	0.14***	0.15***	0.02	–		
7. LS	4.06	1.14	−0.18***	−0.00	0.37***	0.49***	0.10**	0.18***	(0.96)	
8. AS	3.35	0.65	−0.18***	−0.07*	0.37***	0.54***	0.24***	0.10**	0.64***	(0.93)

*p < 0.05, **p < 0.01, ***p < 0.001. Diagonal elements in parentheses represent the Composite Reliability (CR) for the latent constructs derived from the second-order measurement model in Lavaan. Since socio-economic status (SES) is calculated based on a weighted average of parents’ education levels and occupations, the CR value is omitted.

While social comparison was negatively correlated with CSE (*r* = −0.12, *p* < 0.001), it was positively related to students’ PSY (*r* = 0.17, *p* < 0.001), life satisfaction (*r* = 0.10, *p* < 0.01) and academic satisfaction (*r* = 0.24, *p* < 0.001). Socioeconomic status was positively correlated with CSE (*r* = 0.14, *p* < 0.001), PSY (*r* = 0.15, *p* < 0.001), life satisfaction (*r* = 0.18, *p* < 0.001) and academic satisfaction (*r* = 0.10, *p* < 0.01). All the values of CR were within the acceptable range (>0.7).

### 4.3 Common method bias

To mitigate the potential for CMB, we assured the confidentiality and anonymization of the study to respondents in advance. Moreover, the questionnaire was designed to be concise and explicit. Finally, we used Harman’s single-factor test, which indicated that the variance explained by single factor is only 32.1%, well below the suggested threshold of 50%. Finally, we adopted the more rigorous full collinearity assessment approach (Kock, 2015) as integrated in recent SEM studies (Jiang et al., 2025). The results indicated that the Variance Inflation Factors (VIFs) for all principal constructs: CSE (2.19), PSY (2.59), SC (1.26), AS (1.65), and SES (1.03) were well below the threshold of 3.3. These convergent results collectively confirm that CMB does not represent a significant threat to our findings.

### 4.4 Measurement model and confirmatory factor analysis

Following the methodological guidance of Li et al. (2025) on the rigorous assessment of higher-order constructs, we performed a two-stage confirmatory factor analysis (CFA) to justify the hierarchical structure of our instruments. The goodness-of-fit indices indicated an excellent fit for the six-factor solution. The results were as follows: *χ*^2^ = 1710.066, df = 1,634, RMSEA = 0.008, CFI = 0.997, TLI = 0.997, SRMR = 0.022.

Subsequently, we evaluated the reliability and validity of the constructs as shown in Table 2. First, the composite reliability (CR) for higher-order construct ranged from 0.921 to 0.960, exceeding the threshold of 0.7 (Hair et al., 2013). Notably, for the second-order constructs (CSE and PSY), CR and AVE were calculated relative to all underlying indicators to ensure a comprehensive assessment of their hierarchical structure. Convergent validity was supported as all standardized factor loadings were greater than 0.50, and the Average Variance Extracted (AVE) for each construct exceeded the threshold of 0.50 (Hair et al., 2013).

**Table 2:** CR, AVE, and factor loadings.

Construct / Item	Factor loadings
Core self-evaluations (CSE): CR = 0.927, AVE (to all items) = 0.563	
Self-esteem: CR = 0.820, AVE = 0.603	0.962
CSE1	0.769
CSE2	0.784
CSE3	0.776
Generalized self-efficacy: CR = 0.832, AVE = 0.623	0.937
CSE4	0.786
CSE5	0.804
CSE6	0.777
Neuroticism: CR = 0.820, AVE = 0.603	0.972
CSE7	0.779
CSE8	0.772
CSE9	0.779
Locus of control: CR = 0.824, AVE = 0.610	0.974
CSE10	0.781
CSE11	0.764
CSE12	0.798
Psychological capital (PSY): CR = 0.957, AVE (to all items) = 0.535	
Self-efficacy: CR = 0.887, AVE = 0.566	0.975
PSY1	0.753
PSY2	0.756
PSY3	0.783
PSY4	0.760
PSY5	0.716
PSY6	0.741
Hope: CR = 0.881, AVE = 0.553	0.979
PSY7	0.741
PSY8	0.753
PSY9	0.731
PSY10	0.744
PSY11	0.731
PSY12	0.764
Resilience: CR = 0.880, AVE = 0.550	0.975
PSY13	0.731
PSY14	0.709
PSY15	0.755
PSY16	0.752
PSY17	0.750
PSY18	0.751
Optimism: CR = 0.887, AVE = 0.566	0.986
PSY19	0.751
PSY20	0.769
PSY21	0.720
PSY22	0.760
PSY23	0.764
PSY24	0.747
Social Comparison (SC): CR = 0.921, AVE = 0.515	
SC1	0.717
SC2	0.723
SC3	0.736
SC4	0.709
SC5	0.743
SC6	0.712
SC7	0.679
SC8	0.690
SC9	0.728
SC10	0.729
SC11	0.727
Life satisfaction (LS): CR = 0.960, AVE = 0.829	
LS1	0.907
LS2	0.915
LS3	0.907
LS4	0.915
LS5	0.907
Academic satisfaction (AS): CR = 0.933, AVE = 0.664	
AS1	0.795
AS2	0.832
AS3	0.824
AS4	0.824
AS5	0.784
AS6	0.809
AS7	0.835

Finally, discriminant validity was confirmed using the Fornell and Larcker (1981) criterion. As shown in Table 3, the square root of the AVE for each construct was greater than its highest correlation with any other construct.

**Table 3:** Maximum shared variance (MSV) and Fornell–Larcker criterion.

Variable	Square root of AVE	1	2	3	4	5
1. CSE	0.750		0.611***	−0.116**	0.366***	0.366***
2. PSY	0.732	0.611***		0.171***	0.490***	0.540***
3. SC	0.718	−0.116**	0.171***		0.103**	0.241***
4. LS	0.910	0.366***	0.490***	0.103**		0.640***
5. AS	0.815	0.366***	0.540***	0.241***	0.640***	

*p < 0.05, **p < 0.01, ***p < 0.001.

### 4.5 Mediational analysis

We first tested the direct effects between CSE and both life and academic satisfaction. The output indicated a significant direct effect of CSE and life satisfaction [95% CI: (0.014, 0.209)], but an insignificant direct effect of CSE and academic satisfaction [95% CI: (−0.039, 0.153)]. We then tested the indirect effects between CSE and both life and academic satisfaction via PSY using the bootstrapping technique. The bootstrapped confidence intervals of both life and academic satisfaction did not contain zero, indicating significant indirect effects between CSE and both life and academic satisfaction via PSY. These results suggested that PSY partially mediates the relationship between CSE and Generation Z’s life satisfaction, and fully mediates the relationship between CSE and Generation Z’s academic satisfaction. Consequently, hypothesis 1 and hypothesis 2 were both fully supported. The overall testing models were presented in Tables 4, 5.

**Table 4:** Direct effect (*n* = 753).

Path	β	95% CI [LL, UL]
CSE – PSY	0.611***	[0.560, 0.659]
PSY – Life Satisfaction	0.426***	[0.319, 0.518]
CSE – Life Satisfaction	0.106*	[0.014, 0.209]
CSE – PSY	0.611***	[0.560, 0.659]
PSY – Academic Satisfaction	0.519***	[0.402, 0.613]
CSE – Academic Satisfaction	0.054	[−0.039, 0.153]

*p < 0.05, **p < 0.01, ***p < 0.001, CI, confidence interval; LL, lower level; UL, upper level; lower and upper bound of 95% BC bootstrap confidence interval for that effect using 5,000 bootstrap samples.

**Table 5:** Mediational analysis (*n* = 753).

CSE – PSY – Life satisfaction	Estimate	95% BC Bootstrapped CI [LL, UL]	Result
Total effects	0.366***	[0.291, 0.440]	Significant
Direct Effects	0.106*	[0.014, 0.209]	Significant
Indirect Effects	0.260***	[0.198, 0.321]	Significant

CSE – PSY – Academic satisfaction	Estimate	95% BC Bootstrapped CI [LL, UL]	Result
Total effects	0.371***	[0.293, 0.445]	Significant
Direct Effects	0.054	[−0.041, 0.150]	Insignificant
Indirect Effects	0.317***	[0.251, 0.382]	Significant

*p < 0.05, **p < 0.01, ***p < 0.001, CI, confidence interval; LL, lower level; UL, upper level; lower and upper bound of 95% BC bootstrap confidence interval for that effect using 5,000 bootstrap samples.

### 4.6 Moderation analysis

As predicted in Hypothesis 3a and 4a, the relationship between CSE and PSY is moderated by social comparison and socioeconomic status. The result related to hypothesis 3a is listed in Table 6, suggesting a significant interaction of CSE and social comparison, with a negative beta coefficient (*β* = −0.052, *p* < 0.01). The result related to hypothesis 4a is listed in Table 7, suggesting a significant interaction of CSE and socioeconomic status, with a positive beta coefficient (*β* = 0.029, *p* < 0.05).

**Table 6:** Results of regression analysis for moderation by social comparison.

Predictor variables	*β*	*t*
Step 1: Control variables		
Gender	−0.191	−4.473***
Age	−0.004	−0.110
Δ*R*^2^	0.026	
Δ*F*	10.003***	
Step 2: Main effects		
Gender	−0.550	−1.655
Age	0.280	1.113
CSE	0.567	22.628***
SC	0.231	8.591***
Δ*R*^2^	0.410	
Δ*F*	271.382***	
Step 3: Interactions		
Gender	−0.067	−2.042
Age	0.032	1.267
CSE	0.568	23.043***
SC	0.259	9.592***
CSE X SC	−0.052	−5.207***
Δ*R*^2^	0.020	
Δ*F*	27.112***	
*R* ^2^	0.455	
Adj. *R*^2^	0.452	
*F*	124.891***	

*p < 0.05, **p < 0.01, ***p < 0.001. CSE and SC were mean-centered for all analysis.

**Table 7:** Results of regression analysis for moderation by socioeconomic status.

Predictor variables	*β*	*t*
Step 1: Control variables		
Gender	−0.191	−4.473***
Age	−0.004	−0.110
Δ*R*^2^	0.026	
Δ*F*	10.003***	
Step 2: Main effects		
Gender	−0.085	−2.452*
Age	0.022	0.811
CSE	0.532	20.331***
SES	0.002	1.998*
Δ*R*^2^	0.357	
Δ*F*	216.557***	
Step 3: Interactions		
Gender	−0.086	−2.478*
Age	0.022	0.830
CSE	0.530	20.320***
SES	0.002	1.870*
CSE X SES	0.029	2.241*
Δ*R*^2^	0.004	
Δ*F*	5.021*	
*R* ^2^	0.387	
Adj. *R*^2^	0.383	
*F*	94.428***	

*p < 0.05, **p < 0.01, ***p < 0.001. CSE and SES were mean-centered for all analysis.

**Figure 2:**
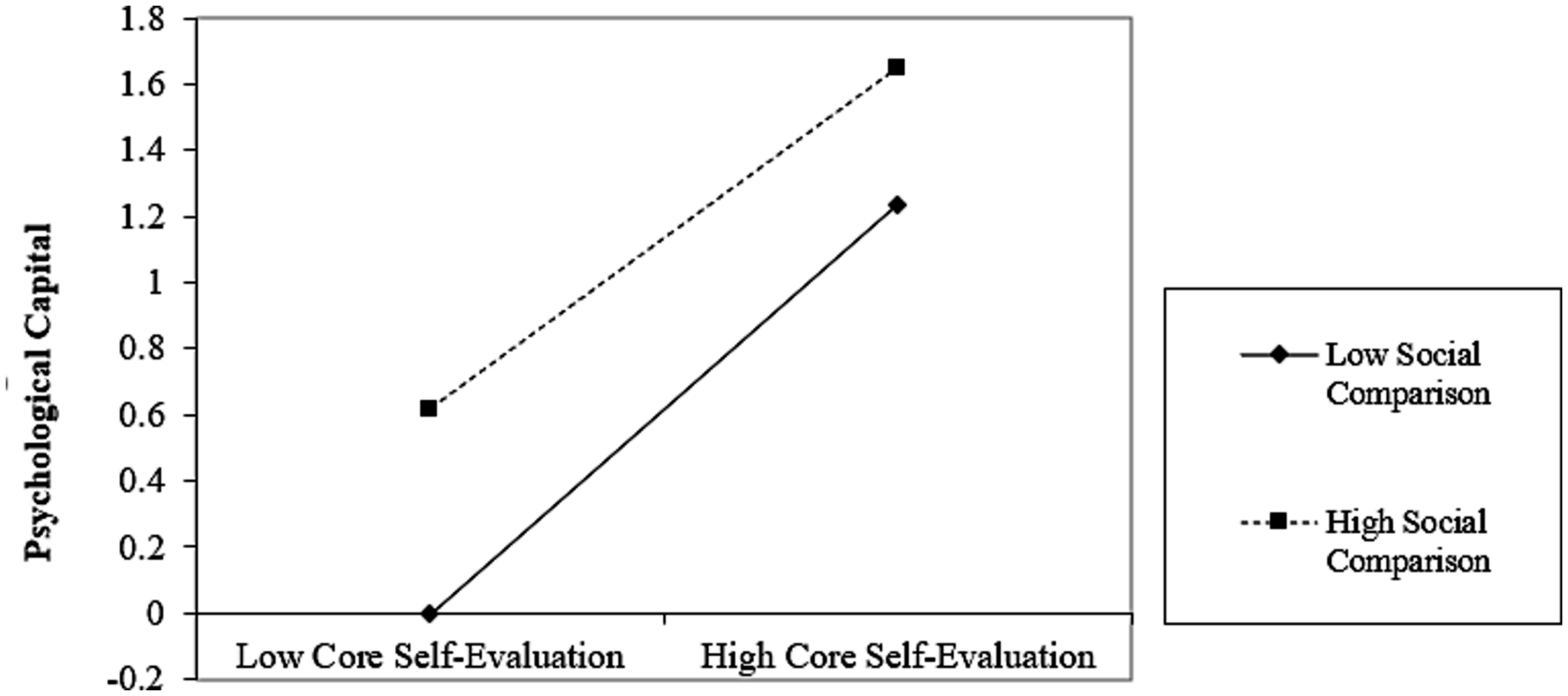
Core self-evaluations and psychological capital by social comparison.

**Figure 3:**
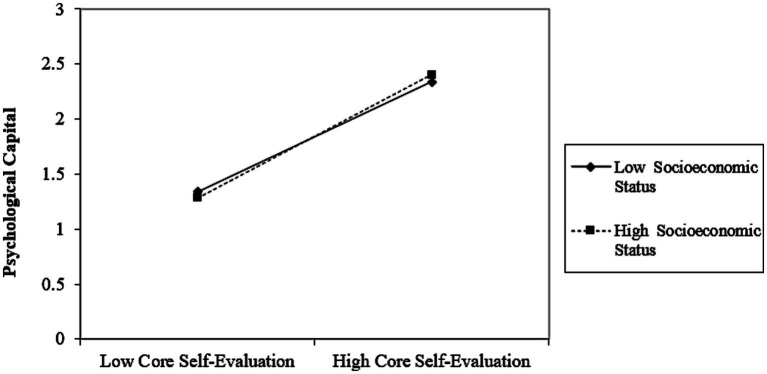
Core self-evaluations and psychological capital by socioeconomic status.

To further understand the abovementioned interaction effects, interaction plots were depicted in Figures 2, 3. Figure 2 presents that the positive relationship between CSE and PSY was relatively weaker while the social comparison was stronger. While Figure 3 presents that the positive relationship between CSE and PSY was relatively stronger as socioeconomic status was higher. Therefore, Hypothesis 3a and 4a were supported.

### 4.7 Double-moderated mediation analysis

At last, we applied PROCESS Model 9 (Hayes, 2015) in SPSS to examine the moderated mediation effects. Table 8 shows the index of the moderated mediation effects and Table 9 shows the double conditional indirect effects of CSE on life and academic satisfaction, suggesting the following:

**Table 8:** Indices of moderated mediation.

Indices of moderated mediation				
Path	Social comparison			
	Index	BootSE	BootLLCI	BootULCI
CSE – PSY – LS	−0.2012	0.0538	−0.3163	−0.1071
CSE – PSY – AS	−0.1368	0.0345	−0.2137	−0.0767

Path	Socioeconomic status			
	Index	BootSE	BootLLCI	BootULCI
CSE – PSY – LS	0.0040	0.0020	0.0003	0.0079
CSE – PSY – AS	0.0027	0.0013	0.0002	0.0053

**Table 9:** Double conditional indirect effect of CSE on life and academic satisfaction (LS, life satisfaction; AS, academic satisfaction) at specific levels of social comparison (SC) and socioeconomic status (SES).

Variable						BC 5000 BOOT	
SC	SES	IND		SE		[LL95, UL95]	
		LS	AS	LS	AS	LS	AS
LOW	LOW	0.6132	0.4169	0.0948	0.0566	[0.4334, 0.8028]	[0.3081, 0.5318]
LOW	MEAN	0.6668	0.4534	0.0953	0.0562	[0.4814, 0.8542]	[0.3446, 0.5651]
LOW	HIGH	0.7204	0.4898	0.1025	0.0609	[0.5221, 0.9233]	[0.3703, 0.6115]
MEAN	LOW	0.5160	0.3509	0.0766	0.046	[0.3690, 0.6674]	[0.2611, 0.4434]
MEAN	MEAN	0.5696	0.3873	0.0759	0.0448	[0.4193, 0.7140]	[0.2975, 0.4750]
MEAN	HIGH	0.6232	0.4238	0.0837	0.0499	[0.4571, 0.7864]	[0.3247, 0.5199]
HIGH	LOW	0.4188	0.2848	0.0639	0.0398	[0.2963, 0.5466]	[0.2080, 0.3643]
HIGH	MEAN	0.4724	0.3212	0.0617	0.0375	[0.3455, 0.5902]	[0.2462, 0.3935]
HIGH	HIGH	0.5260	0.3577	0.0698	0.0427	[0.3838, 0.6591]	[0.2736, 0.4398]

Coefficients represent specific indirect effects and standard errors at different values of both moderators, SC and SES.

The indirect effect of CSE on life satisfaction via PSY was moderated by social comparison (index of moderated mediation = −0.2012, bootstrapping 95%CI [−0.3163, −0.1071]).

The indirect effect of CSE on academic satisfaction via PSY was also moderated by social comparison (index of moderated mediation = −0.1368, bootstrapping 95%CI [−0.2137, −0.0767]).

The indirect effect of CSE on life satisfaction via PSY was moderated by socioeconomic status (index of moderated mediation = 0.004, bootstrapping 95%CI [0.0003, 0.0079]).

The indirect effect of CSE on academic satisfaction via PSY was moderated by socioeconomic status (index of moderated mediation = 0.0027, bootstrapping 95%CI [0.0002, 0.0053]).

Therefore, H3b, H3c, H4b, H4c were supported.

Moreover, Hayes and Scharkow (2013) suggested the conditional indirect effect could be fully proved if the interactions were significant and the confidence intervals did not contain zero. In this study, the moderating effects of both social comparison and socioeconomic were significant on the indirect effect of CSE and life/academic satisfaction via PSY. More specifically, the indirect effects on life satisfaction were strongest when socioeconomic status was at its highest level and social comparison was at its lowest level. On the contrary, the indirect effects on academic satisfaction were strongest when socioeconomic status was at its highest level and social comparison was at its lowest level.

**Figure 4:**
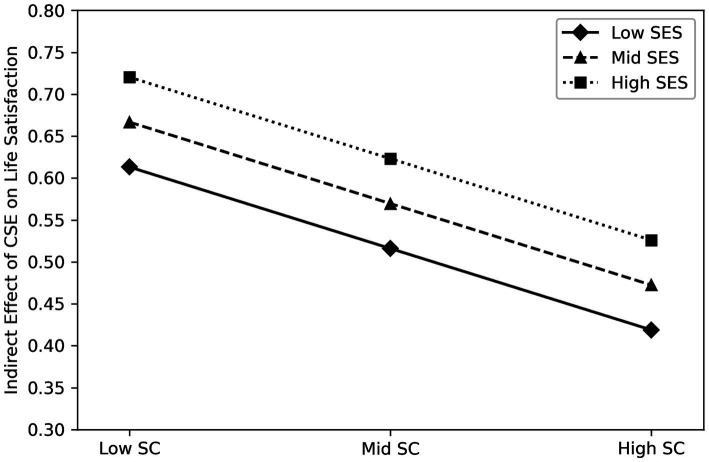
Indirect effect of CSE on life satisfaction via psychological capital.

**Figure 5:**
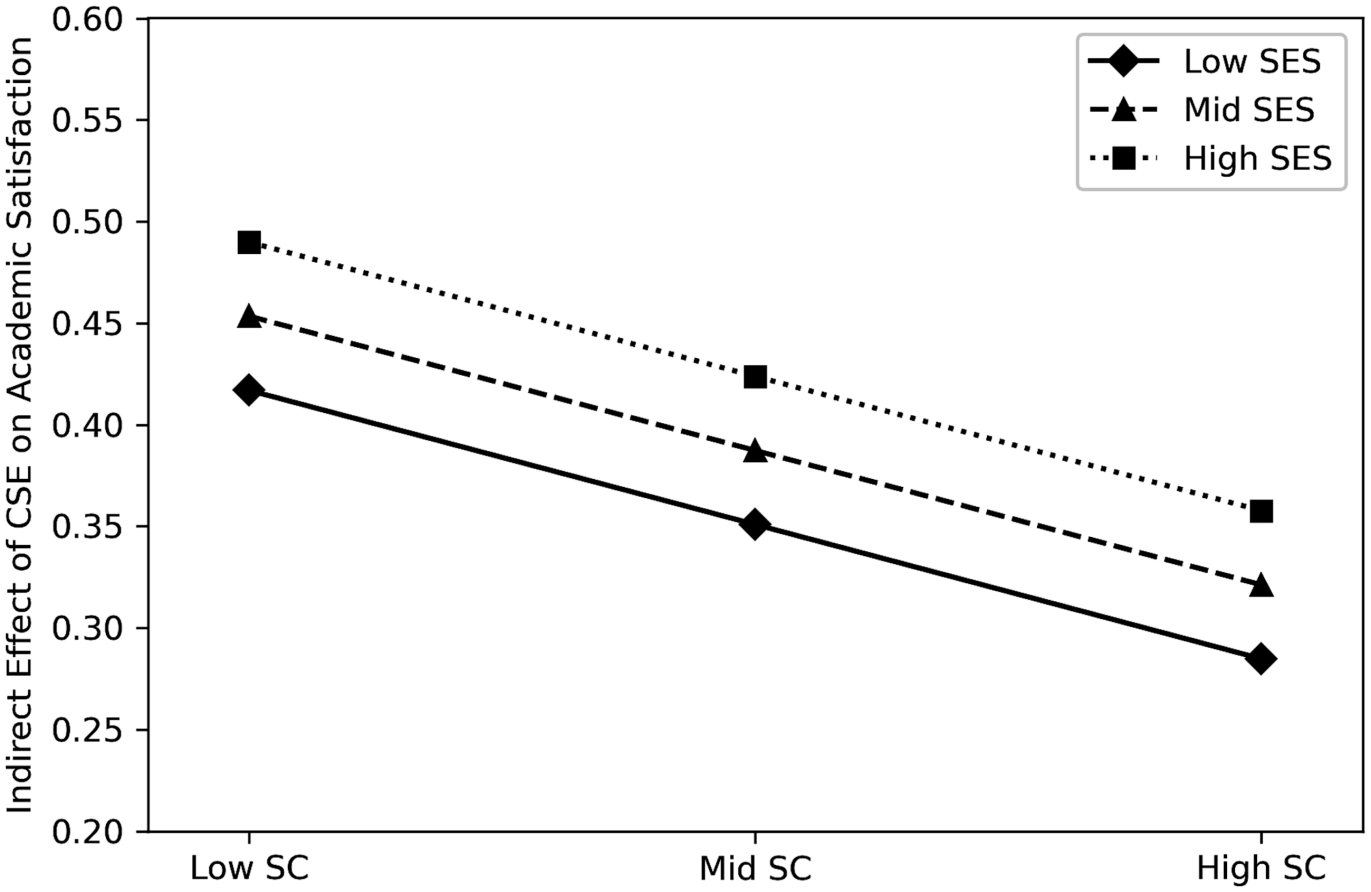
Indirect effect of CSE on academic satisfaction via psychological capital.

The indirect effect of CSE on life and academic satisfaction via PSY was described using the conditional indirect effect graph in Figures 4, 5. In summary, a lower level of social comparison was linked with a stronger indirect effect from CSE to life and academic satisfaction through PSY. On the contrary, a higher level of socioeconomic status was associated with a stronger indirect effect from CSE to life and academic satisfaction through PSY.

**Figure 6:**
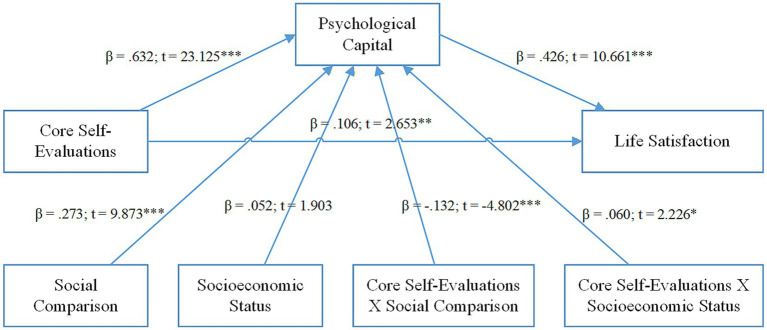
Examination of moderated mediation on life satisfaction: standardized path coefficients (*p < 0.05, **p < 0.01, ***p < 0.001).

**Figure 7:**
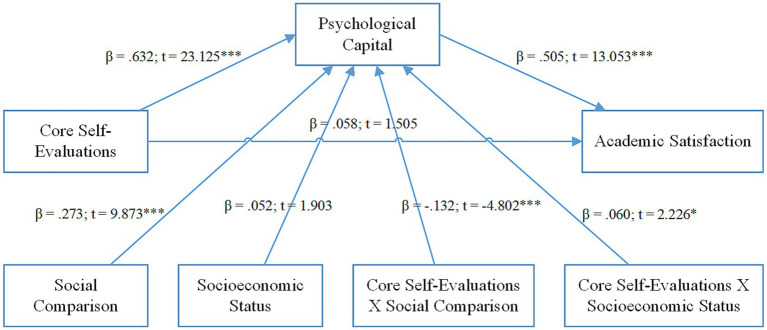
Examination of moderated mediation on academic satisfaction: standardized path coefficients (*p < 0.05, **p < 0.01, ***p < 0.001).

Finally, we performed a full path analysis using the lavaan package in R, incorporating both the measurement model and the structural model to evaluate the robustness of our findings. As shown in Figures 6, 7, while the point estimates slightly differ from the OLS-based results due to the inclusion of latent constructs, the direction and significance of all paths remain highly consistent. These results further confirm that Hypotheses 1, 2, 3, and 4 are all supported.

## 5 Discussion

The first and foremost task in our study was to exam how CSE relates to the satisfaction of Generation Z. After analyzing the data from 753 Chinese students, we found that social comparison, socioeconomic status, and PSY have different effects on the relation between CSE and Generation Z’s satisfaction.

In support of Positive Psychology, our findings emphasize the critical role of PSY during the post-epidemic era, which could determine students’ resistance against setbacks in such a special environment. Base on Social Comparison Theory and Social Cognitive Theory, we also proposed how social comparison and socioeconomic status could influence our hypotheses separately.

In particular, our study found that while social comparison was negatively correlated with CSE (*r* = −0.12, *p* < 0.001), it was positively related to students’ PSY (*r* = 0.17, *p* < 0.001), life satisfaction (*r* = 0.10, *p* < 0.01), and academic satisfaction (*r* = 0.24, *p* < 0.001). The findings suggested that although social networking apps (Facebook, WeChat) have provided platforms for social comparison, which could hurt students’ CSE, those apps did not decrease students’ life or academic satisfaction. To a certain extent, making downward comparisons could make students more optimistic about the future, thus improving their PSY, life satisfaction, and academic satisfaction. This finding also complements the research of Buunk et al. (2001), which suggested that downward social comparison could improve the feeling of satisfaction in intimate relationships to some extent, as well as further confirm the research of Buunk and Ybema (2003) about people’s complex emotions of “feeling bad, but satisfied.”

Moreover, previous research indicated that socioeconomic status influences one’s pessimism and optimism (Heinonen et al., 2006), which was a portion of PSY. Our findings suggested that students with higher socioeconomic status may have better CSE and PSY in times of difficulties, as higher socioeconomic status could improve one’s self-esteem, hope, and optimism, thereby improving one’s life and academic satisfaction.

The results verified that the indirect effect of CSE on life and academic satisfaction through PSY for Generation Z was strongest when socioeconomic status was at its highest level and social comparison was at its lowest level. These findings could offer a few contributions to theory and practice.

### 5.1 Theoretical implications

In support of Positive Psychology, we contributed a new perspective for both CSE and PSY. Firstly, we confirmed the previous studies about CSE’s essential role, such as CSE is important for one’s satisfaction (Smedema et al., 2021), well-being (Chen et al., 2022), and academic record (Laguerre et al., 2023). In the meanwhile, our findings echoed most previous studies’ views about the crucial role of PSY by demonstrating that PSY has influenced one’s satisfaction (Culbertson et al., 2010). Particularly, our findings suggested that PSY also has a great influence on the academic satisfaction of Generation Z.

Based on the above-mentioned findings, we further explored the relationship between CSE and PSY. Recent studies have paid little attention to the relationship between them except a few studies (Firouznia et al., 2021; Paloș, 2023). By answering calls in this literature (Howard, 2017), our model extended a new area in the empirical test of the relationship between CSE and PSY. According to our findings, students with better CSE might have higher self-confidence, which could lead to stronger PSY. Based on our study, researchers could investigate further into how to help Generation Z improving their life and academic satisfaction by strengthening their CSE and PSY.

Interestingly, our study contributed a new perspective to Social Comparison Theory. This study increased the precision of the theory, on account of finding how social comparison played a critical role in understanding how CSE affect Generation Z’s satisfaction via PSY. Beyond merely viewing the relationship between social comparison and satisfaction (Ruggieri et al., 2021), our study adds new knowledge to Social Comparison Theory by combined social comparison with PSY, CSE, life satisfaction, and academic satisfaction. Our study offered a new research avenue focusing on the boundary condition of social comparison on mediation rule of PSY in the CSE – life satisfaction and CSE – academic satisfaction links, indicating that a lower level of social comparison was associated with a stronger indirect effect from CSE to life and academic satisfaction through PSY. The result of our study could help to resolve previous arguments about the relationship between social networking apps and social comparison, as well as enrich the social comparison literature in the aspect of personal social life (Buunk et al., 2007).

Furthermore, our results added to the paucity of literature on Social Cognitive Theory. On the one hand, from the views of social cognitive theory, personal behavior is greatly influenced by environmental factors. Therefore, we address socioeconomic status as a moderator. The moderated mediation exam indicated that the indirect effects on academic satisfaction were strongest when socioeconomic status was at its highest level, which highlights the importance of socioeconomic status. Our study not only confirmed traditional researches about the positive influence of socioeconomic status on personal satisfaction but also advanced the consequence by combining socioeconomic status with CSE and PSY, which were not studied by antecedences. On the other hand, while self-efficacy is a portion of both CSE and PSY, our study extended and portrayed a new understanding of Self-Efficacy Theory by investigating and highlighting the relationship between the two, about which few empirical studies have been done.

After analyzing the moderating effect of social comparison, this study deepened the understanding that Generation Z, who is proficient in social networking apps (Turner, 2015). As the online learning scheme more or less affected Generation Z’s academic satisfaction, we also gain a deeper awareness of successfully that the usage of internet flipped classrooms may provide higher quality of educational resources and greater academic satisfaction to Generation Z.

To sum up, we provided a theoretical and empirical verification for both how and why CSE relates to the life and academic satisfaction of Generation Z.

### 5.2 Practical implications

Beyond the theoretical aspect, our findings also hold significant practical implications. In the post-epidemic era, the high-paying opportunities have decreased due to the economic downturn, the transformation of social concepts, and the inequality of educational opportunities have brought strong psychological gaps and dissatisfaction to students of Generation Z. Hence, our findings provided a deeper understanding into the pivotal role of CSE. It supported the propositions students with better CSE were more confident in the post-epidemic era, they may have a better locus of control and self-efficacy, which lead to stronger PSY. Moreover, students who were more confident may have a higher level of life and academic satisfaction.

As a representative social group in mainland China, college students may suffer from potential academic pressure. In particular, our findings paid more attention to the essentials of PSY. In other words, PSY fully mediates the relationship between CSE and Generation Z’s academic satisfaction. This phenomenon means that, on one hand, students should be assisted to better develop their abilities coping with stressors or changes. On the other hand, teachers and parents should be more aware of the importance of students’ psychological adjustment during post-epidemic era, rather than one-sided pursuing school enrollment. Although some students might be addicted to mobile games and do nothing productive while staying at home, parents should set a good example for their children by establishing a regular daily life and communicating with their children more. As an engineer of the human soul, teachers should pay more attention to students’ physical and mental health and guide their students to focus on the positive sides of society rather than panic or misleading information. Students should strengthen the scientific knowledge of self-protection and learn the scientific methods for psychological adjustment. While it is possible for people to have negative emotions at times during this special period, the government should provide psychological assistance hotlines to help citizens with serious psychological issues.

## 6 Conclusion

The life and academic satisfaction of Generation Z is attracting greater theoretical and empirical attention from scholars in the present age. Our study indicated that low social comparison and high socioeconomic status could strengthen the positive effect of CSE on Generation Z’s satisfaction in daily life. Especially, CSE and PSY play essential roles in students’ mental state. Understanding how CSEs influences the life and academic satisfaction of Generation Z via PSY could shed light on the way in which we could promote the physical and intellectual integrity of the Chinese youth in the long run.

### 6.1 Limitations and recommendations for future studies

First, this study launched an investigation on Generation Z in Southeast China and most of the respondents were college students owing to time and resource constraints. The results of this article can only represent a part of Chinese college students. Future studies may expand this research framework in various countries and regions, conducting a cross-region or cross-cultural survey.

Another limitation of our study is the single source of data, resulting in the CMV in our study. To handle this problem, students were assured anonymity and confidentiality of their answers, avoiding the existence of social desirability bias. Moreover, we checked the questionnaire repeatedly which was designed concisely and clearly. Then we assessed CMV with the Harmon 1-factor test and full collinearity assessment approach, which indicated that it was not a serious problem. However, the potential for such bias could be further minimized if future research adopts longitudinal designs or incorporates multiple data sources.

Finally, our study opened up interesting and new routes for the future. Our moderated mediation model in this study was tested for Generation Z. As each generation has its own unique characteristic, future studies could extend longitudinal researches for Generation X, Generation Y, and the millennial generation.

## Data Availability

The original contributions presented in the study are included in the article/supplementary material, further inquiries can be directed to the corresponding author.
